# Improving Ethyl Acetate Production in* Baijiu* Manufacture by* Wickerhamomyces anomalus* and* Saccharomyces cerevisiae* Mixed Culture Fermentations

**DOI:** 10.1155/2019/1470543

**Published:** 2019-01-13

**Authors:** Guangsen Fan, Chao Teng, Dai Xu, Zhilei Fu, Pengxiao Liu, Qiuhua Wu, Ran Yang, Xiuting Li

**Affiliations:** ^1^Beijing Advanced Innovation Center for Food Nutrition and Human Health, Beijing Technology & Business University (BTBU), Beijing 100048, China; ^2^School of Food and Chemical Engineering, Beijing Technology and Business University (BTBU), Beijing 100048, China; ^3^Beijing Engineering and Technology Research Center of Food Additives, Beijing Technology & Business University (BTBU), Beijing 100048, China

## Abstract

Ethyl acetate content has strong influence on the style and quality of* Baijiu*. Therefore, this study investigated the effect of* Saccharomyces cerevisiae* Y3401 on the production of ethyl acetate by* Wickerhamomyces anomalus* Y3604. Analysis of cell growth showed that Y3401 influences Y3604 by nutrient competition and inhibition by metabolites, while the effect of Y3604 on Y3401 was mainly competition for nutrients. Mixed fermentation with two yeasts was found to produce more ethyl acetate than a single fermentation. The highest yield of ethyl acetate was 2.99 g/L when the inoculation ratio of Y3401:Y3604 was 1:2. Synergistic fermentation of both yeasts improved ethyl acetate production and increased the content of other flavor compounds in liquid and simulated solid-state fermentation for* Baijiu*.* Saccharomyces cerevisiae* had a positive effect on ethyl acetate production in mixed culture and provides opportunities to alter the aroma and flavor perception of* Baijiu*.

## 1. Introduction

*Baijiu* is normally made from sorghum alone or from a mixture of wheat, corn, peas, millet, rice, and sorghum by solid-state fermentation. It is generally considered the national alcoholic beverage of China, and it is a typical example of a traditional Chinese fermented food [1]. More than 1870 compounds have been identified in* Baijiu*, and many studies have shown that the quality and style of* Baijiu* are determined by these flavoring compounds [1]. Among these compounds, ethyl acetate has been identified as one of the main aroma components [2]. It is the only chemical that is used in the national standards as a clear indicator of style and quality of beverage for almost all types of* Baijiu*. It is especially important for the formation of particular* Baijiu* styles, including strong flavor, light flavor, rice flavor, and Xifeng flavor products. Ethyl acetate is one of the four main esters in strong flavor* Baijiu*, and it is the main fragrance in light flavor* Baijiu* [2].

It is well known that microbial metabolism is the main source of ethyl acetate in* Baijiu* manufacture. There are many biological strains in the* Baijiu* fermentation process that can produce ethyl acetate, including yeasts, bacteria, and molds [3, 4]. Studies have shown that the aroma-producing yeasts (also named ester-producing yeasts), a kind of non-*Saccharomyces* wild yeast, are the main strains that produce ethyl acetate [5]. These yeasts can improve the fragrance of the main body of* Baijiu* and can be used to actively change its flavor [6]. Because of the special contribution to the flavor quality of* Baijiu*, the aroma-producing yeasts can be called “flavor regulators” [7]. Coincidentally, non-*Saccharomyces* yeasts are now generally regarded as having the ability to improve wine or beer characteristics, such as complexity, mouthfeel, and integration of flavors [8, 9]. Although there has been limited research on the roles of non-*Saccharomyces* yeasts in* Baijiu* production, many producers have extensive practical knowledge of these aroma-producing yeasts and how to use them to achieve improved sensory effects in* Baijiu*. In view of these considerations, it is beneficial to isolate, study, and apply the aroma-producing yeasts, especially those with high-yield ethyl acetate production characteristics. This approach is of great significance in efforts to improve the quality of* Baijiu*.

At present, our research team has access to aroma-producing yeast Y3604, identified as* Wickerhamomyces anomalus*, which is reported to be among the highest strains for ethyl acetate producing microorganisms [2]. When Y3604 was inoculated in sorghum hydrolysate medium (SHM) with the addition of 4% anhydrous ethanol and 0.1% acetic acid, the total ethyl acetate production was 16.92 g/L [2]. It is noteworthy that the concentration of ethyl acetate produced by Y3604 was less than 2 g/L when ethanol was not added as a substrate [2]. Thus, ethanol is an important substrate for the synthesis of ethyl acetate by yeast esterification [10]. Although Y3604 produces a small amount of ethanol in the process of growth and metabolism, it is insufficient for the synthesis of ethyl acetate.

It is well known that* Baijiu* fermentation occurs in a spontaneous process and multiple species are involved [1]. Microbial communities are the major drivers of fermentation processes and may include mold, bacteria, yeast, Actinomycetes, and Archaea. These organisms perform metabolic processes that can include decomposition, synthesis, and transformation and thereby produce* Baijiu* with many flavor substances [11, 12]. While it is apparent that there is no lack of functional microorganisms to produce ethanol in* Baijiu* brewing, the most important strain for ethanol production is* Saccharomyces cerevisiae* [13].

Given that* S. cerevisiae* is the most important ethanol-producing strain and* W. anomalus* is a high-yielding ethyl acetate strain, a study of the interaction of these strains should help to improve the content of ethyl acetate in the fermentation system and have significant implications for improving* Baijiu* quality. This study has examined the interaction of* S. cerevisiae* and* W. anomalus* with a view to tuning the fermentation and enhancing the aroma and ester content. This research will lay a solid foundation for the application of both yeasts in* Baijiu* production.

## 2. Materials and Methods

### 2.1. Screening for High-Yielding Ethanol Yeasts

Yeasts were screened from* Daqu*, which was provided by Gujinggong Liquor (strong flavor, Anhui Gujing Group) and Laobaigan (light flavor, Hengshui Laobaigan), according to the method described previously [2]. Each isolated colony was inoculated to a yeast extract peptone dextrose (YPD) medium (glucose 20 g, peptone 20 g, yeast extract 10 g, agar powder 20 g, and ddH_2_O 1000 mL, pH 6.0–6.2, autoclaved at 115°C for 20 min) in a slant tube and numbered. A loop of the yeasts stored in each slant tube after initial screening was inoculated into 50 mL of alcoholic fermentation medium (glucose 200 g, yeast extract 10 g, peptone 20 g, ammonium sulfate 1 g, monopotassium phosphate 1 g, magnesium sulfate 1 g, and ddH_2_O 1000 mL, pH 5.8–6.0, autoclaved at 115°C for 20 min) with an initial number of colonies of 1.0 × 10^6^ CFU/mL (static culture, 30°C, 72 h). At the end of the fermentation, the medium was ultracentrifuged at 10,000 rpm for 10 min. After filtration, the supernatants were analyzed for ethanol by high-performance liquid chromatography (HPLC). The yeast that produced more ethanol was selected.

### 2.2. Identification of Yeast

The screened yeast was identified by morphological observation, physiological and biochemical characteristics, and phylogenetic analysis according to Fan et al. [2].

### 2.3. Microbial Interaction

Fermentations were carried out for the single cultures of Y3401 and Y3604, respectively, as well as for their mixed culture. Y3401 and Y3604 were precultured, respectively, in a YPD liquid medium at 30°C for 24 h. Cells were collected by centrifugation and washed with saline. Using SHM as the fermentation medium, cells were inoculated to obtain a cellular population of 1 × 10^6^ CFU/mL. A 1:1 mixed-culture fermentation of Y3401 and Y3604 was tested. The SHM was prepared according to the methods described by Fan et al. [2]. Aliquots of 50 mL of SHM were fermented in 250-mL flasks at 30°C with shaking (180 rpm) for 6 days. Three flasks were randomly selected each day throughout the fermentation to determine yeast cell growth, fermenting property, and pH.

### 2.4. Evaluation of Effects of Cell-Free Culture Filtrates

Cell-free culture filtrates were prepared from cultures that used conditions described by Kato et al. [14]. Separate cultures of Y3401 and Y3604 were cultivated in SHM with stirring at 180 rpm at 30°C for 3 days. The supernatant of the culture solution was collected by centrifugation, and the cell-free culture filtrate (CFCF) was prepared by filter sterilization with 0.22-*μ*m-pore filters. Because the CFCF was considered to lack some nutrients, SHM-filtrate medium was used for the culture experiment. A 1:1 mixture of SHM and the cell-free culture filtrate (SHM-CFCF) was used, or 800 g/L glucose (SHM-CFCF-G) was added to this mixture to adjust the initial sugar concentration. Each precultured isolate was inoculated into the SHM-filtrate medium to obtain a final cell count of 1 × 10^6^ CFU/mL. Growth was then measured as the optical density reading at 560 nm (OD_560_) after cultivation with stirring at 180 rpm at 30°C for 24 h. Growth in the SHM-filtrate medium was compared with that in the control medium (1:1 mixture of SHM and uninoculated medium used for the filtrate preparation described above). The culture experiments were carried out in triplicate.

### 2.5. Effect of Inoculation Method on Ethyl Acetate Content

Triplicate fermentations were carried out in 250-mL Erlenmeyer flasks containing 50 mL of SHM at 30°C for 6 days after inoculation with precultures of Y3401 or Y3604. Simultaneous mixed fermentation was performed by inoculating 1 × 10^6^ CFU/mL of Y3401 and Y3604 (SMF). One sequential mixed fermentation used an initial inoculation of 1 × 10^6^ CFU/mL of Y3401, followed 12 h later with 1 × 10^6^ CFU/mL of Y3604 to form the yeast mixture (S-W). The other sequential mixed fermentation reversed this process, using an initial inoculation of 1 × 10^6^ CFU/mL of Y3604, which was followed 12 h later with 1 × 10^6^ CFU/mL of Y3401 to give the mixture (W-S). Single-culture fermentations by Y3604 (W) and Y3401 (S) were also carried out under the same conditions. All of the above described fermentations were carried out in static culture as per the* Baijiu* production process. Three flasks were randomly selected each day throughout the fermentation process to determine the remaining reducing sugars, fermenting property, ethanol content, ethyl acetate content, and flavor compounds (fermentation finish).

### 2.6. Effect of Inoculation Ratio on Ethyl Acetate Content

Mixed-culture fermentations with Y3401:Y3604 ratios of 1:1, 3:1, 6:1, 1:2, and 1:3 were tested. Y3401 and Y3604 were simultaneously inoculated into SHM and the final cell population of Y3604 was 1 × 10^6^ CFU/mL at the beginning of the fermentation. All of these fermentations were also carried out in static culture. The remaining reducing sugars, fermenting property, ethanol content, ethyl acetate content, and flavor compounds (fermentation finish) were determined.

### 2.7. Simulated Solid-State Fermentation for Baijiu

Simulated solid-state fermentation, prepared as in previous studies, was performed to study the effect of interactions between Y3401 and Y3604 on the ethyl acetate content in* Baijiu* [15]. Four separate batches were prepared by simultaneously adding precultured Y3401 and Y3604 to light flavored* Daqu* in the following combinations: A, Y3401 + Y3604; B, Y3401 + Y3604 +* Daqu*; C,* Daqu*; D, not inoculated.* Daqu* (Hengshui Laobaigan) was ground and inoculated into batches B and C (at 12.5% w/w). Y3401 and Y3604 were separately precultured to a final cell population of 1 × 10^6^ CFU/mL, and then 10 mL of each yeast culture was simultaneously inoculated into batches A and B at the beginning of the fermentation. The fermentations were carried out in static conditions for 30 days at room temperature. Flavor compounds were analyzed by headspace solid-phase microextraction gas chromatography–mass spectrometry (HS-SPME GC–MS). All fermentation experiments were conducted in triplicate.

### 2.8. Analytical Methods

Yeast cell growth during fermentation was obtained by viable cell quantification using the classical plate count method. Samples were taken aseptically throughout the fermentations and diluted appropriately with saline. YPD agar plates were used for enumeration of the yeasts. The fermenting property of Y3401 and Y3604 in the fermentation process was measured using the carbon dioxide (CO_2_) weight loss method [16]. Mass loss caused by CO_2_ evolution was monitored by weighing the fermentation flasks every day. The pH was determined with a pH meter. Remaining reducing sugars were measured by the dinitrosalicylic acid (DNS) assay and ethanol content was determined by HPLC using a BioRad 87H column and a refractive index detector (Varian 355 RI) according to the method of Meng et al. [12]. Ethyl acetate content was determined by GC–MS and flavor compounds were analyzed by HS-SPME GC–MS, following previously described methods [2, 17].

### 2.9. Statistical Analysis

Each treatment was performed in triplicate. All statistical analyses were performed with SPSS16.0 (SPSS, Chicago, IL, USA) and OriginPro 9.1 (OriginLab, Northampton, MA, USA). Analysis of variance (ANOVA) was used to compare the means. Mean separations were performed by Duncan's multiple range tests. Differences at* P *< 0.05 were considered significant.

## 3. Results and Discussion

### 3.1. Screening and Identification of High-Yielding Ethanol Yeast

A total of 46 yeasts were isolated and screened for ethanol yield from* Daqu*. Among all the strains, only 12 yeasts produced more than 10 g/L of ethanol. One isolate (Y3401) achieved high ethanol production (70 g/L) and was selected for further study (Table 1). Initially, strain Y3401 was preliminarily identified by morphology and microstructure. Its colonies on Wallerstein laboratory nutrient agar culture medium (WL; glucose 50 g, yeast extract 4 g, tryptone 5 g, monopotassium 0.55 g, potassium chloride 0.425 g, calcium chloride 0.125 g, magnesium sulfate 0.125 g, ferric chloride 0.0025 g, manganese sulfate 0.0025 g, agar 20 g, bromocresol green 0.022 g, and dd H_2_O 1000 mL, pH 6.5, autoclaved at 115°C for 20 min) were yellow-green centered with a yellow edge, 2–3 mm in diameter, opaque, raised, and sticky, with a wet, smooth surface and inerratic edges. Under a high-magnification zoom lens (10 × 40), morphological characterization of Y3401 showed that cells were ellipsoidal, occurring as a single cell or as parental bud pairs, and asexual budding reproduction occurred at the ends of the cells. Then strain Y3401 was identified based on physiological and biochemical tests. It was observed to ferment glucose and sucrose, but not maltose, galactose, raffinose, lactose, or trehalose. In carbohydrate utilization screening, glucose, sucrose, succinic acid, ethyl alcohol, glycerol, gluconic acid, ribose, and lactic acid were positive as sole carbon sources, but sorbitol, xylose, rhamnose, erythritol, d-(+)-gluconic acid *δ*-lactone, and methanol were negative. Ammonium sulfate, potassium nitrate, ethylamine, and l-lysine were utilized as sole nitrogen sources for growth, but potassium nitrite and creatinine were not utilized. Further identification was confirmed by 26S rDNA sequencing and analysis. Strain Y3401 was confirmed as* S. cerevisiae* based on the similarity of results observed in BLAST analysis and phylogenetic analysis, and the sequence was submitted to GenBank (Supplementary Figure S1, NCBI accession no. MG548387). It was deposited in the China General Microbiological Culture Collection Center (CGMCC) under accession no. 14828.

### 3.2. Influence of Microbial Interaction on Cell Growth

**Figure 1:**
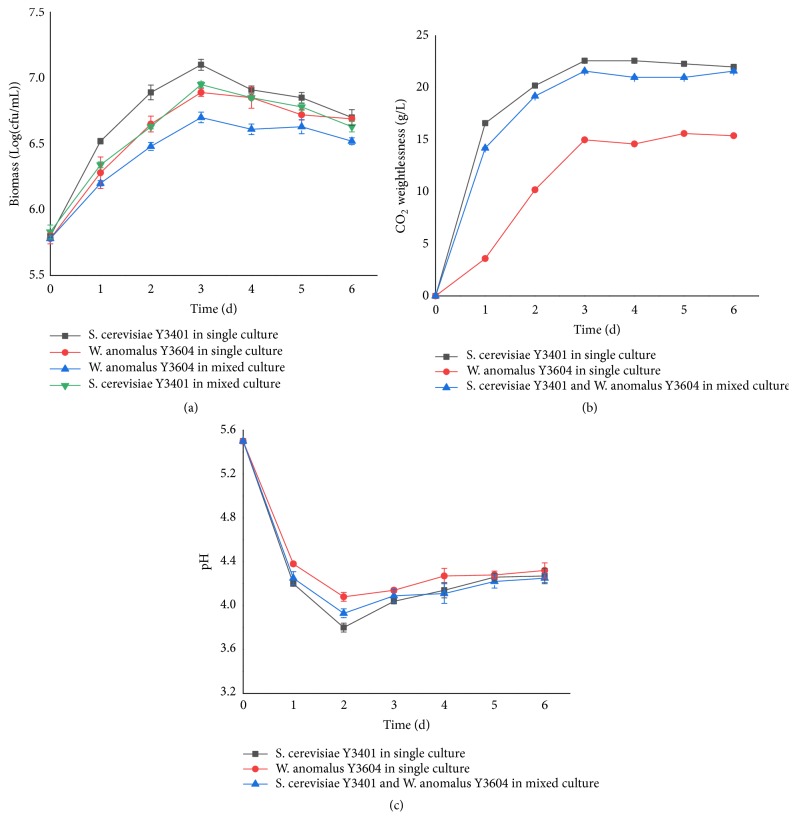
Cell growth, fermenting property and pH in single and mixed cultures of* S. cerevisiae* Y3401 and* W. anomalus* Y3604 with the inoculation ratio of 1:1. (a), Cell growth of* S. cerevisiae* Y3401 and* W. anomalus* Y3604 in single and mixed cultures, black square,* S. cerevisiae* Y3401 in single culture, red circle,* W. anomalus* Y3604 in single culture, green triangle down,* S. cerevisiae* Y3401 in mixed culture, blue triangle up,* W. anomalus* Y3604 in mixed culture; (b), Fermenting property of* S. cerevisiae* Y3401 and* W. anomalus* Y3604 in single and mixed cultures, black square,* S. cerevisiae* Y3401 in single culture, red circle,* W. anomalus* Y3604 in single culture, blue triangle up,* S. cerevisiae* Y3401 and* W. anomalus* Y3604 in mixed culture; (c), pH of* S. cerevisiae* Y3401 and* W. anomalus* Y3604 in single and mixed cultures, black square,* S. cerevisiae* Y3401 in single culture, red circle,* W. anomalus* Y3604 in single culture, blue triangle up,* S. cerevisiae* Y3401 and* W. anomalus* Y3604 in mixed culture. Results are the average and bars indicate the SD.

Cell growth, fermenting property, and pH were monitored in single and mixed cultures.  Figure 1(a) shows viable cells of Y3401 and Y3604 in single and mixed cultures. In all cultures tested, the growth trends of both yeasts were similar. Both yeasts reached the maximum population within 3 days and then decreased slightly as fermentation progressed. It is important to mention that both yeasts exhibited a lower growth rate in coculture and the final biomass was lower than in the respective pure cultures (Figure 1(a)). The results demonstrated that the growth of each yeast in mixed culture was influenced by the other [18, 19]. This result concurs with a previous report in which there was growth between* S. cerevisiae *and* W. anomalus*, and this fact is evident in other studies for other non-*Saccharomyces* yeasts where population is controlled during the fermentation [20–22]. It is also noteworthy that the growth rate and biomass of Y3401 were faster and higher than Y3604 in either single or mixed culture. However, some studies have shown that* W. anomalus* grows better than* S. cerevisiae*, which may be a result of different strains [23]. The maximum population of Y3401 and Y3604 reached 1.3 × 10^8^ CFU/mL and 7.7 × 10^7^ CFU/mL in single cultures and 8.9 × 10^7^ CFU/mL and 5.0 × 10^7^ CFU/mL in mixed cultures, respectively. Given that* S. cerevisiae* is known to preferentially utilize reducing sugars to proliferate rapidly, it appears to have competed with* W. anomalus* for the carbon source, resulting in slow growth of* W. anomalus* and a disparity in cell number for the two yeasts in coculture condition [18].

Carbon dioxide weight loss is commonly used to reflect the fermenting property of yeast in fermentation or during the* Baijiu* brewing process [24]. As shown in Figure 1(b), the fermenting property of Y3401 was superior to that of Y3604, being in accordance with the results of Medina et al. [25]. We also noticed that the fermenting property of the mixed-strain fermentation was similar to that of Y3401, and it had an advantage over that of Y3604. Thus, it can be inferred that Y3604 has little effect on the fermenting property of Y3401, and Y3401 improved the fermenting property of the mixed culture. In this respect, it is feasible to produce more ethyl acetate by Y3604 by using the ethanol, as the precursor for ethyl acetate, produced by Y3401. In fact, it is generally believed that non-*Saccharomyces* can inhibit the fermenting property of* S. cerevisiae* [19, 25]. Mixed-culture fermentation using* S. cerevisiae* and non-*Saccharomyces* resulted in sluggish fermentation, compared with fermentations using pure cultures of* S. cerevisiae* [25]. In our study, it was found that there were some effects in the early stage of coculture. However, in the late stage of fermentation, the fermenting property in the coculture system was gradually restored to that of Y3401 in single culture because of the increase in the number of* S. cerevisiae*. This result is in accordance with observations of* S. cerevisiae* and* Kloeckera apiculate* and is evident in other studies of other non-*Saccharomyces* yeasts [21, 26].

The pH was monitored during the fermentations, as shown in Figure 1(c). In single culture, the pH decreased quickly in the first 2 days, from 5.50 to 3.80 for Y3401 and from 5.50 to 4.08 for Y3604, and then increased slowly to a final pH of 4.27 and 4.32, respectively. The pH of the mixed culture showed a trend similar to that of the single cultures, especially Y3401. In the early stage of fermentation, the yeast formed acidic substances such as acetic acid, resulting in a decrease in pH [26]. After initial breeding, the yeast entered the stage of alcohol fermentation, which led to a constant rise of alcohol concentration. The yeast would be expected to secrete proteases at this stage, hydrolyzing protein to amino acids, which would then degenerate to produce ammonia. The formation of ethanol and NH_3_ resulted in an increase in the pH [18]. This result was in agreement with previous reports[18, 26], and, in a previous study, we found that Y3604 had good pH tolerance and was able to grow at pH 2.0. Furthermore, acidic conditions are favored for production of ethyl acetate by Y3604 [2]. Therefore, we speculate that the decrease of pH caused by the growth of Y3401 would not be an adverse influence on Y3604 to produce ethyl acetate.

### 3.3. Influence of Cell-Free Culture Filtrate on Cell Growth

During mixed fermentations, several factors can affect the growth of strains, such as competition for nutrients (mainly carbon sources), the presence of toxic compounds, low available oxygen conditions, high ethanol concentration, cell-cell contact, and quorum sensing [20, 27]. There was a certain degree of interaction between Y3401 and Y3604 in mixed culture and the cell population of both reached a maximum after 3 days, which means that nutrients or metabolites in the medium after 3 days became a limiting factor for yeast growth. Therefore, we briefly analyzed the interaction of both strains from the perspectives of carbon source and metabolites using the CFCF from day 3. As shown in Table 2, the growth of Y3401 was affected by the carbon source (A1 and A2 in Table 2) and was weakly affected by metabolites of Y3604 (A3 and A4 in Table 2). The metabolites of Y3401 had an inhibitory effect on the growth of Y3604 (B1 and B2 in Table 2), which is consistent with previous reports [23]. By analysis of results B3 and B4, it appears that the growth of Y3604 was inhibited by its own metabolites. These results were consistent with the findings of a previous study of the interaction between* S. cerevisiae* and* Pichia anomala* in coculture [18]. In other previous studies of mixed culture fermentations, yeasts were found to produce other metabolites besides ethanol, such as short- to medium-chain fatty acids or peptides, which can become inhibitory to other yeast species [28–30]. Production of these metabolites varies significantly with yeast species and strain [30], while the metabolites also exhibit different fungistatic effects against different strains [31]. These results have special significance for optimizing the mixed fermentation of both strains to improve ethyl acetate content.

### 3.4. Optimization of Mixed Fermentations for Ethyl Acetate Yield

The previous study gave us a preliminary understanding of the interaction between Y3401 and Y3604. Next, we studied how the strains cofermented to improve the content of ethyl acetate with different inoculation methods and inoculation ratios in static cultures. In addition to focusing on the content of ethyl acetate, we also analyzed reducing sugars, fermenting property, ethanol content, and flavor components after fermentation. Thus, a comprehensive evaluation was conducted for both yeasts in the cofermentation from multiple perspectives.

#### 3.4.1. Effect of Inoculation Method on Ethyl Acetate Content

The utilization of reducing sugar is an important index for judging the growth of yeast [18]. Interestingly, significant difference was observed in reducing sugar consumption between the single and mixed fermentations (Supplementary Figure S2(a)). The results showed that fermentation went to completion for all fermentations, and, at day 3, the mixed fermentation systems and the Y3401 single culture contained less than 10 g/L reducing sugar. In other words, in single culture of Y3604, the rate of reducing sugar consumption was lower than that of Y3401 or the mixed culture. Two possible explanations were proposed: first, the inoculum size in single culture was smaller than that in mixed culture; second, Y3401 uses sugar faster than Y3604. At the same time, it was apparent that earlier inoculation of Y3401 saw faster utilization of sugar in the mixed fermentation system, and* S. cerevisiae* showed the highest fermentability in single culture in accordance with previous reports [18, 20]. We concluded that Y3604 had little or no effect on the utilization of sugar by Y3401. This further indicates from another perspective that Y3604 had limited effect on the growth of Y3401. Similar results for other non-*Saccharomyces* have been reported previously [18, 20, 27].

In analyzing the fermentations, Supplementary Figure S2(b) shows that the curve profile of fermenting property was the opposite to that for the reducing sugars, although the internal laws reflected by both curves were basically the same. The results showed that the fermenting property of Y3401 was superior to Y3604. The fermenting property in the mixed fermentation system, which was inoculated with Y3401, was higher than that in a single system of* W. anomalus*, and the fermenting property was related to the time of inoculation of Y3401. In general, earlier inoculation of Y3401 gave higher fermentation capacity, especially in the first 2 days. These results are basically in line with the results of previous reports [18, 32]. The above interpretations of the changes in reducing sugars also applied to the fermenting property. At the same time, the fermenting property of all fermentation systems decreased gradually with time and was very low after 3 days. This may have been caused by strong consumption of nutrients, especially reducing sugars, and the accumulation of metabolites in the fermentation system, resulting in a decrease in cell growth.

The results for ethanol analysis are illustrated in Supplementary Figure S3(c). It is apparent that Y3604 was able to produce ethanol in static culture, while the yield was less than that of Y3401. This observation is similar to reports of other non-*Saccharomyces* [23, 33, 34]. For mixed fermentations, the amount of ethanol produced was higher than that in single culture of Y3604, implying that Y3401 increased the amount of ethanol in the fermentation system. Literature reports suggest that ethanol production is significantly increased with* S. cerevisiae* inoculation in mixed fermentations and that the lowest ethanol levels are seen in the pure cultures of non-*Saccharomyces* [20, 32, 35]. For inoculation timing, earlier inoculation of Y3401 gave the higher yields of ethanol, in agreement with some previous reports [32, 36]. In summary, Y3401 was able to provide ethanol, which is a precursor of ethyl acetate, for Y3604 to produce more ethyl acetate. Medina et al. [25] reported a significant increase in flavor compounds, such as ethyl acetate, by cofermentation of* S. cerevisiae* with non-*Saccharomyces *in wine.

**Figure 2:**
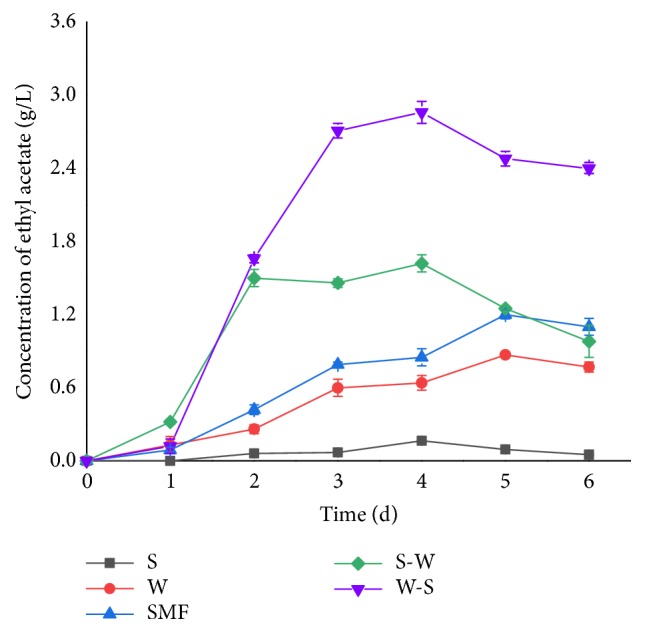
The change of concentration of ethyl acetate in different fermentation with different inoculation methods. Black square, single-culture fermentation by* S. cerevisiae* Y3401 (S); red circle, single-culture fermentation by* W. anomalus* Y3604 (W); blue triangle up, simultaneous mixed fermentation was performed by inoculating 1×10^6^ CFU/mL of* S. cerevisiae* Y3401 and* W. anomalus* Y3604 (SMF); green diamond, inoculating 1×10^6^ CFU/mL of* S. cerevisiae* Y3401 for 12 h firstly, then 1×10^6^ CFU/mL of* W. anomalus* Y3604 was added (S-W); violet triangle down, inoculating 1×10^6^ CFU/mL of* W. anomalus* Y3604 for 12 h firstly, then 1×10^6^ CFU/mL of* S. cerevisiae* Y3401 was added (W-S). Results are the average and bars indicate the SD.

In previous reports, the use of a non-*Saccharomyces*–*S. cerevisiae* couple was found to significantly boost the production of most detected compounds, more particularly in higher alcohols, esters, acids, and terpenes, while there are few reports on the optimal conditions for increasing the yield of ethyl acetate in mixed fermentation [32, 35, 37–40]. Ethyl acetate production was different among different culture fermentation systems in our study (Figure 2). Mixed culture produced more ethyl acetate than single culture during fermentation, and ethyl acetate production was higher in mixed fermentation systems with sequential inoculation than in simultaneous mixed fermentation. Among the mixed fermentations, W-S was the best method of inoculation for ethyl acetate production with 2.86 g/L, which was 3.28 times the level achieved by single culture of Y3604. Although ethanol production in S-W and SMF was slightly higher than that in W-S, both produced less ethyl acetate than W-S. This may have been caused by rapid growth of Y3401, which was more vigorous than Y3604, in the S-W and SMF fermentations, resulting in nutrient competition and metabolite inhibition to Y3604 [20, 26, 41].

The results of HS-SPME GC–MS analysis of flavor fractions are shown in Supplementary Table S1. Significant differences between culture types were observed for all the aroma compounds analyzed. It was found that mixed fermentations showed higher content of flavor compounds than single fermentation by Y3604, especially for esters, ethanol, and higher alcohols. A previous study indicated that mixed fermentations of* S. cerevisiae* and* W. anomalus* show interesting oenological properties and can provide a favorable combination for production of esters and linear alcohols [38]. Because of the inoculation of Y3401 in mixed fermentation, ethanol content increased significantly when compared with single fermentation. This result also led to a significant rise in ethanol conversion to ethyl acetate. Thus, the presence of* S. cerevisiae* in the mixed fermentations significantly increased the ethyl acetate production [20]. In addition, levels of phenethyl alcohol and the corresponding phenethyl acetate with a rose-like odor were higher in mixed fermentation than in single fermentation. Ye et al. [20] also reported that the simultaneous mixed culture of* S. cerevisiae* and* W. anomalus* demonstrated an increase in phenethyl acetate content. It is also noteworthy that a few flavor compounds, such as caprylic acid and ethyl caprylate, were produced in mixed fermentation because of the inoculation of Y3401; these compounds were not present in the single fermentation by Y3604. Previous reports showed that the presence and persistence of* S. cerevisiae* and its metabolic interactions with* W. anomalus* in the mixed fermentations influenced the production of volatile compounds [20, 42].

#### 3.4.2. Effect of Inoculation Ratio on Ethyl Acetate Content

Overall, the reducing sugars were almost fully consumed within 3 days in all mixed fermentations, and the consumption rate of reducing sugars increased as the proportion of Y3401 increased (Supplementary Figure S3(a)). In addition, the consumption of reducing sugars was similar when the inoculation ratios of Y3401:Y3604 were 6:1 and 3:1. These ratios also gave the fastest consumption of reducing sugars. The utilization of reducing sugars was about the same for inoculation ratios of 1:1 and 1:2. These results are consistent with a previous finding that showed that the consumption rate of reducing sugars was higher when the proportion of* S. cerevisiae* was higher [26]. In accordance with the above results, the growth rate of Y3401 was higher than that of Y3604.

Supplementary Figure S3(b) shows that when Y3401 was dominant, the fermenting property was better than when Y3604 was dominant. Among the mixed cultures, the fermenting property was highest when the inoculation ratio of Y3401:Y3604 was 3:1. It is worth noting that the lowest fermenting property was observed for a Y3401:Y3604 ratio of 1:1. This was somewhat different from the trend of reducing sugars consumption, perhaps because the interaction between them was most obvious in this case, and some nutrients, including reducing sugars, were consumed and converted to antimicrobial metabolites in the course of interaction [23, 43, 44].

The yield of ethanol usually reached the highest level on the third or fourth day, and the content of ethanol decreased slightly in the later stages of fermentation, because the rate of ethanol synthesis was lower than the rate of its consumption and conversion to other substances (Supplementary Figure S3(c)). In addition, Supplementary Figure S3(c) shows that ethanol production increased with the increasing proportion of Y3401. Generally, compared with Y3604 in single fermentation, the ethanol content increased when Y3401 was cocultured with Y3604, which introduced a large number of precursors for the synthesis of ethyl acetate (Supplementary Figure S2(c); Figure S3(c)) [18, 32].

**Figure 3:**
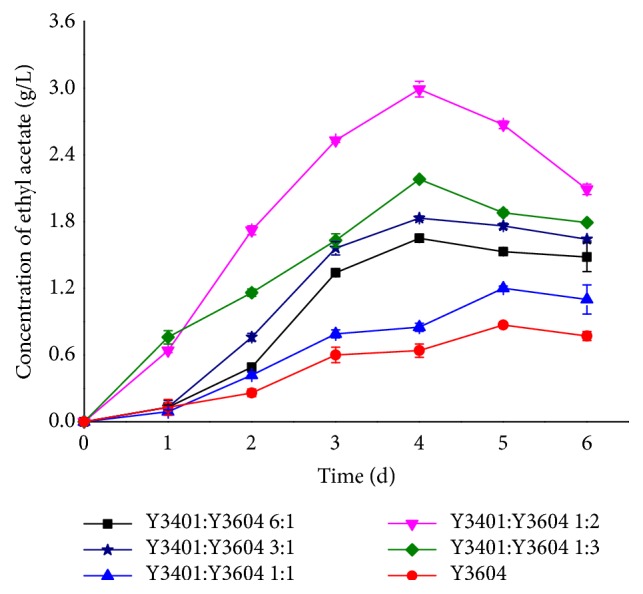
The change of concentration of ethyl acetate in different fermentation with different inoculation ratio. Black square, inoculation ratio of* S. cerevisiae* Y3401 and* W. anomalus* Y3604 is 6:1; green star, inoculation ratio of* S. cerevisiae* Y3401 and* W. anomalus* Y3604 is 3:1; blue triangle up, inoculation ratio of* S. cerevisiae* Y3401 and* W. anomalus* Y3604 is 1:1; purple triangle down, inoculation ratio of* S. cerevisiae* Y3401 and* W. anomalus* Y3604 is 1:2; green diamond, inoculation ratio of* S. cerevisiae* Y3401 and* W. anomalus* Y3604 is 1:3; red circle, single-culture fermentation by* W. anomalus* Y3604. Results are the average and bars indicate the SD.

Figure 3 shows that the amount of ethyl acetate increased first and then decreased with time for the different inoculation ratios. The yield of ethyl acetate was higher when Y3604 was dominant in mixed fermentation, while the yield was the least when the two yeasts were in equal proportion. When Y3604 was dominant, the highest ethyl acetate yield was 2.99 g/L when the inoculation ratio of Y3401:Y3604 was 1:2. When Y3401 was dominant, the higher ethyl acetate yield was obtained when the ratio of Y3401:Y3604 was 3:1. These results may be explained by several factors. Although Y3604 has high ethanol tolerance, its growth and metabolism would always be inhibited under high ethanol concentration, which was the case with Y3401 [20, 45]. The metabolites of Y3401 are also known to have some effect on Y3604 [26, 27, 41]. Therefore, the higher yield of ethyl acetate occurred when Y3604 was the predominant strain because more substrate ethanol would be produced by Y3401. However, when the proportion of Y3604 was too high, it inevitably had some influence on Y3401 and affected its ability to provide ethanol, which is one of the precursors of ethyl acetate [20, 45]. Similarly, when Y3401 was the predominant strain, although more ethanol was generated, the synthesis of ethyl acetate by Y3604 was affected because the normal metabolism of Y3604 was suppressed by high ethanol [20, 45]. Therefore, in this case, the higher yield of ethyl acetate occurred at a relatively low Y3401:Y3604 ratio of 3:1. It is noteworthy that when the cultures were inoculated in equal proportions, the yield of ethyl acetate may have been lower because of more intense interaction.

The levels of volatile compounds for different inoculation ratios are shown in Supplementary Table S2. Except for ethanol, phenethyl alcohol, and ethyl acetate, the differences between the flavor compounds were minor for the different inoculation ratios. Ethanol and ethyl acetate levels were basically consistent with the previous results. The change of phenethyl alcohol content was similar to that of ethanol, and the content of phenethyl alcohol increased as the proportion of Y3401 increased. The content of phenethyl alcohol in the mixed fermentation system was higher than that in single fermentation by each of the yeasts, which means that the yeasts showed a synergistic effect in the production of phenethyl alcohol (Supplementary Tables S1 and S2). This result is similar to previous reports [46].

### 3.5. Simulated Solid-State Fermentation for Baijiu with Y3401 and Y3604

At present,* Baijiu *production is carried out as a solid-state fermentation [15, 43]. In using the Y3401 and Y3604 strains to enhance the taste of* Baijiu*, we also studied the flavor compounds associated with these two yeasts in the simulated solid-state fermentation for* Baijiu*. Analysis revealed (Table 3) that many flavor compounds were present in the simulated solid-state fermentation, including alcohols, esters, ketones, acids, and alkanes. The combination of these substances determines the quality of* Baijiu*. The highest abundance of flavor compounds occurred in experiment B (Y3401/Y3604/*Daqu*). It is considered that isoamyl alcohol, ethyl phenylacetate, ethyl tetradecanoate, ethyl 9-hexadecenoate, ethyl palmitate, ethyl oleate, styrene, 1-caryophyllene, and pentadecane are probably related to the metabolisms of Y3401 and Y3604, while the formation of ethyl isovalerate, ethyl pentadecanoate, isovaleric acid, and phenylacetaldehyde are probably related to the interaction of both yeasts and the microorganisms provided by* Daqu*. The results show that the flavor profile of* Baijiu* can be altered by the addition of extrinsic microbes. This effect was not caused by the flavor production ability of the extrinsic microbes, but by the interaction between the extrinsic and intrinsic microbes [23]. When compared with experiment C (*Daqu*), experiments A (Y3401/Y3604) and B (Y3401/Y3604/*Daqu*) showed significant increases in ethyl acetate, phenethyl alcohol, and methyl catechol, which have significant effects on the quality of* Baijiu*. Thus, it is apparent that the synergistic effect of Y3401 and Y3604 can improve esterification and enhance the flavor of* Baijiu*.

## 4. Conclusion

It is well known that* Baijiu* prepared by spontaneous fermentation does not always meet the expectations of consumers. Although the use of additional inoculated functional microbes is an attractive way to improve* Baijiu* quality, the microbial interactions are poorly understood, and the use of selected microbes does not always have a positive result. This work investigated the microbial interactions between* S. cerevisiae* and* W. anomalus* as two functional microbes. We found that, under suitable culture conditions,* S. cerevisiae* can enhance the production of ethyl acetate by* W. anomalus* during* Baijiu* fermentation. The highest yield of ethyl acetate was 2.99 g/L in SHM medium with a Y3401:Y3604 inoculation ratio of 3:1. This process of tuning mixed fermentations for* Baijiu* production can be used to improve product quality and complexity to ultimately produce beverages with distinctive sensory properties.

## Figures and Tables

**Table 1:** The ethanol yields for each yeast.

No.	Ethanol yield (g/L)	No.	Ethanol yield (g/L)
Y3401	70	Y3506	27
Y3402	20	Y3604	13
Y3403	35	Y3215	24
Y3501	12	Y1105	47
Y3502	10	Y1801	26
Y3503	24	Y1502	31

**Table 2:** Growth suppression by addition of cell free filtrate.

No.	Culture medium^*A*^	A	B
		Relative value (*S. cerevisiae* Y3401)	Relative value (*W. anomalus* Y3604)
1	SHM-CFCF-Y3401	0.91±0.02^a^	0.74±0.05^a^
2	SHM-CFCF-G-Y3401	0.99±0.02^b^	0.75±0.04^a^
3	SHM-CFCF-Y3604	0.93±0.01^a^	0.82±0.02^b^
4	SHM-CFCF-G-Y3604	0.97±0.05^ab^	0.85±0.02^b^

^*A*^  SHM-CFCF-Y3401: 1:1 mixture of SHM and the cell-free culture filtrate of *S. cerevisiae* Y3401; SHM-CFCF-G-Y3401: Adding glucose into 1:1 mixture of SHM and the cell-free culture filtrate of *S. cerevisiae* Y3401; SHM-CFCF-Y3604: 1:1 mixture of SHM and the cell-free culture filtrate of *W. anomalus* Y3604; SHM-CFCF-G-Y3604: Adding glucose into 1:1 mixture of SHM and the cell-free culture filtrate of *W. anomalus* Y3604.

Note: Same lowercase letters in each column do not differ significantly at 5% probability by Duncan's multiple range tests.

**Table 3:** The volatile compounds in the different simulated solid-state fermentations (*μ*g/kg).

**Volatile compounds**	**A**	**B**	**C**	**D**	**Odor thresholds (**μ**g/L)**
Ethanol	521±42^a^	723±71^b^	535±112^a^	150±37^a^	100,000
Isoamyl alcohol	27±12^a^	133±21^b^	-	-	179,190.83
*β-*Phenethyl alcohol	695±131^c^	3,436±243^d^	53±13^b^	19±1^a^	28,922.73
1-Octen-3-ol	12±11^a^	115±22^b^	236±91^c^	732±244^d^	6.12
2-Phenyl-propan-2-ol	-	166±92^b^	35±11^a^	-	/
Σ **Higher alcohols**	**1,255**	**4,573**	**859**	**901**	
Ethyl acetate	833±91^c^	625±72^b^	64±13^a^	-	32,551.60
Ethyl caprylate	57±12^a^	76±32^a^	45±21^a^	-	12.87
Ethyl caprate	-	23±1^a^	22±2^a^	-	1122.30
Ethyl phenylacetate	39±26^a^	182±81^b^	-	-	406.83
Ethyl isovalerate	-	73±27	-	-	6.89
Dimethyl phthalate	9±7^a^	18±3^a^	23±11^a^	-	/
Ethyl tetradecanoate	34±9^a^	87±26^b^	-	-	800
Dibutyl phthalate	64±8^a^	322±105^b^	-	-	/
Ethyl pentadecanoate	-	87±23	-	-	7,000
Ethyl 9-hexadecenoic acid	9±7^a^	88±37^b^	-	-	1,500
Ethyl palmitate	584±107^a^	1,632±209^b^	-	-	2,000
Ethyl oleate	587±104^a^	1,563±212^b^	-	-	/
Σ **Esters**	**2,216**	**4,776**	**154**	-	
2-Octanone	583±109^bc^	861±232^c^	464±68^ab^	288±99^a^	/
Acetophenone	157±87^b^	36±27^a^	-	-	/
3-Octanone	-	62±17^b^	33±8^a^	143±18^c^	/
Σ **Ketones**	**740**	**959**	**497**	**431**	
Methyl catechol	981±32^b^	2,361±192^c^	-	57±37^a^	23
4-Vinylguaiacol	2,153±128^b^	94±47^a^	87±21^a^	-	1,100
4-Ethylguaiacol	-	11,609±296^b^	23±16^a^	64±28^a^	33
Σ **Phenols**	**3,134**	**14,064**	**110**	**121**	
Isooctanoic acid	13±1^a^	21±16^a^	11±9^a^	-	/
Isovaleric acid	-	93±17	-	-	1045.47
Σ **Acids**	**13**	**114**	**11**	-	
Azulene	109±28^b^	253±57^c^	23±2^a^	127±69^b^	/
Styrene	23±8^a^	114±27^b^	-	-	80
1-Caryophyllene	114±37^a^	108±67^a^	-	-	/
Σ **Alkenes**	**246**	**475**	**23**	**127**	
Pentadecane	32±8^a^	44±27^a^	-	74±36^a^	/
Hexadecane	23±0^a^	52±12^b^	18±17^a^	-	300,000-400,000
Eicosane	9±1^a^	18±11^a^	-	-	/
Σ **Alkanes**	**64**	**114**	**18**	**74**	
Phenylacetaldehyde	-	22±1^a^	13±0^a^	-	5.2
**Sum**	**7,668**	**25,075**	**1,672**	**1,654**	

A: Only *S. cerevisiae* Y3401 and *W. anomalus* Y3604 were in the solid-state fermentations system; B: *S. cerevisiae* Y3401, *W. anomalus* Y3604 and *Daqu* were in the solid-state fermentations system; C, Only *Daqu* was in the solid-state fermentations system; D, no inoculation was in the solid-state fermentations system.

Note: Data are average of three replicates ± standard deviations; “-”, not detected; Same lowercase letters in each line do not differ significantly at 5% probability by Duncan's multiple range tests; “/”, not available.

## Data Availability

The data used to support the findings of this study are available from the corresponding author upon request.

## Supplementary Materials

Supplementary Materials Table S1: some of the main volatile compounds at the end of the different fermentations by different inoculation methods. Table S2: some of the main volatile compounds at the end of the different fermentations by different inoculation ratio. Figure S1: neighbor-joining phylogenetic tree based on 26S rDNA gene sequence of strain Y3401 and its closest relative species. Figure S2: the change of remaining reducing sugar, fermenting property, and concentration of ethanol in different fermentation with different inoculation methods. Figure S3: the change of remaining reducing sugar, fermenting property, and concentration of ethanol in different fermentation with different inoculation ratio.
