# Supplementary material for: Improving Ethyl Acetate Production in Baijiu Manufacture by Wickerhamomyces anomalus and Saccharomyces cerevisiae Mixed Culture Fermentations
Source: Biomed Res Int. 2019 Jan 13;2019:1470543. doi: 10.1155/2019/1470543 (PMC6348840; doi:10.1155/2019/1470543)
Supplement: Supplementary Materials — Table S1: some of the main volatile compounds at the end of the different fermentations by different inoculation methods. Table S2: some of the main volatile compounds at the end of the different fermentations by different inoculation ratio. Figure S1: neighbor-joining phylogenetic tree based on 26S rDNA gene sequence of strain Y3401 and its closest relative species. Figure S2: the change of remaining reducing sugar, fermenting property, and concentration of ethanol in different fermentation with different inoculation methods. Figure S3: the change of remaining reducing sugar, fermenting property, and concentration of ethanol in different fermentation with different inoculation ratio. [file 1470543.f1.docx]

**Supplementary Tables**

**TABLE S1:** Some of the main volatile compounds at the end of the different fermentations by different inoculation methods (g/L)

| Volatile compounds | SHM | S | W | SMF | S-W | W-S | Odor thresholds (µg/L) |
| --- | --- | --- | --- | --- | --- | --- | --- |
| Ethanol | - | 4.32±0.07^e^ | 0.5±0.02^a^ | 2.19±0.02^c^ | 3.69±0.09^d^ | 1.68±0.03^b^ | 100,000 |
| β*-*Phenethyl alcohol | - | 1.27±0.12^b^ | 0.86±0.03^a^ | 2.11±0.07^d^ | 2.51±0.11^e^ | 1.51±0.20^c^ | 28,922.73 |
| Isoamyl alcohol | - | 0.28±0.09^a^ | 0.85±0.12^c^ | 0.88±0.08^c^ | 0.97±0.20^c^ | 0.79±0.02^b^ | 179,190.83 |
| Isobutyl alcohol | - | - | 0.09±0.03^a^ | 0.05±0.03^a^ | - | 0.06±0.01^a^ | 40,000 |
| Σ Higher alcohols | **-** | **1.55** | **1.80** | **3.04** | **3.48** | **2.36** | |
| Ethyl acetate | - | 0.03±0.00^a^ | 0.31±0.07^b^ | 0.71±0.13^c^ | 0.75±0.10^c^ | 0.82±0.03^c^ | 32,551.60 |
| Phenethyl acetate | - | 0.05±0.02^a^ | 0.14±0.09^b^ | 0.41±0.13^d^ | 0.27±0.11^c^ | 0.39±0.02^cd^ | 908.83 |
| Isoamyl acetate | - | - | 0.02±0.01^a^ | - | 0.05±0.03^a^ | 0.03±0.02^a^ | 93.93 |
| Ethyl caprylate | - | 0.04±0.03^a^ | - | 0.02±0.01^a^ | 0.04±0.03^a^ | - | 12.87 |
| Σ Esters | **-** | **0.12** | **0.47** | **1.14** | **1.11** | **1.24** | |
| Acetic acid | - | - | 0.07±0.02^a^ | 0.04±0.03^a^ | 0.05±0.02^a^ | 0.03±0.02^a^ | 36 |
| Isobutyric acid | - | - | 0.03±0.02^a^ | - | 0.02±0.01^a^ | - | 2,300 |
| Methylbutanoic acid | - | - | - | - | 0.02±0.02 | - | 1045.47 |
| Octanoic acid | - | 0.01±0.00^a^ | - | 0.03±0.02^ab^ | 0.05±0.03^b^ | 0.01±0.00^a^ | 2701.23 |
| Caproic acid | - | - | - | - | 0.02±0.01 | - | 2517.16 |
| Σ Acids | **-** | **0.01** | **0.10** | **0.07** | **0.16** | **0.04** | |
| Vinyl guaiacol | 0.02±0.01^a^ | - | 0.10±0.02^b^ | 0.06±0.02^b^ | 0.05±0.03^ab^ | - | 209.30 |
| Sum | **0.02** | **6.00** | **2.97** | **6.50** | **8.49** | **5.32** | |

SHM: sorghum hydrolysate medium; S: single-culture fermentation by *S. cerevisiae* Y3401; W: single-culture fermentation by *W. anomalus* Y3604; SMF: simultaneous mixed fermentation was performed by inoculating 1×10^6^ CFU/mL of *S. cerevisiae* Y3401 and *W. anomalus* Y3604; S-W, inoculating 1×10^6^ CFU/mL of *S. cerevisiae* Y3401 for 12 h firstly, then 1×10^6^ CFU/mL of *W. anomalus* Y3604 was added; W-S: inoculating 1×10^6^ CFU/mL of *W. anomalus* Y3604 for 12 h firstly, then 1×10^6^ CFU/mL of *S. cerevisiae* Y3401 was added.

Note: Data are average of three replicates ± standard deviations; “-”, not detected; Same lowercase letters in each line do not differ significantly at 5% probability by Duncan’s multiple range tests.

**TABLE S2:** Some of the main volatile compounds at the end of the different fermentations by different inoculation ratio (g/L)

| Volatile compounds | *S. cerevisiae* Y3401 : *W. anomalus* Y3604 | | | | | |
| --- | --- | --- | --- | --- | --- | --- |
|  | **6:1** | **3:1** | **1:1** | **1:2** | **1:3** | **0:1** |
| Ethanol | 2.37±0.13^c^ | 2.25±0.11^c^ | 2.19±0.02^c^ | 2.24±0.08^c^ | 1.68±0.07^b^ | 0.5±0.02^a^ |
| β*-*Phenethyl alcohol | 2.31±0.14^c^ | 2.24±0.18^c^ | 2.11±0.07^c^ | 1.84±0.09^b^ | 1.83±0.10^b^ | 0.86±0.03^a^ |
| Isoamyl alcohol | 0.87±0.07^a^ | 0.91±0.11^a^ | 0.88±0.08^a^ | 0.85±0.12^a^ | 0.93±0.09^a^ | 0.85±0.12^a^ |
| Isobutyl alcohol | 0.05±0.02^a^ | 0.05±0.03^a^ | 0.05±0.03^a^ | - | 0.07±0.02^a^ | 0.09±0.03^a^ |
| Σ Higher alcohols | **3.23** | **3.20** | **3.04** | **2.69** | **2.83** | **1.80** |
| Ethyl acetate | 0.71±0.04^b^ | 0.82±0.09^b^ | 0.71±0.13^b^ | 1.03±0.05^c^ | 0.91±0.10^bc^ | 0.31±0.07^a^ |
| Phenethyl acetate | - | 0.3±0.02^b^ | 0.41±0.13^b^ | 0.33±0.11^b^ | 0.34±0.05^b^ | 0.14±0.09^a^ |
| Isoamyl acetate | 0.04±0.02^ab^ | 0.03±0.01^a^ | - | 0.06±0.02^b^ | - | 0.02±0.01^a^ |
| Ethyl caprylate | - | 0.03±0.01^a^ | 0.02±0.01^a^ | - | - | - |
| Σ Esters | **0.75** | **1.18** | **1.14** | **1.42** | **1.25** | **0.47** |
| Acetic acid | 0.10±0.02^b^ | 0.03±0.02^a^ | 0.04±0.03^a^ | 0.08±0.04^ab^ | 0.10±0.01^b^ | 0.07±0.02^ab^ |
| Isobutyric acid | 0.02±0.01^a^ | - | - | - | 0.02±0.00^a^ | 0.03±0.02^a^ |
| Methylbutanoic acid | - | - | - | 0.02±0.01 | - | - |
| Octanoic acid | 0.01±0.01^a^ | 0.07±0.02^b^ | 0.03±0.02^ab^ | 0.03±0.00^a^ | 0.02±0.01^a^ | - |
| Caproic acid | - | 0.02±0.00 | - | - | - | - |
| Σ Acids | **0.13** | **0.12** | **0.07** | **0.13** | **0.14** | **0.10** |
| Vinyl guaiacol | 0.06±0.01^a^ | 0.06±0.03^ab^ | 0.06±0.01^a^ | 0.07±0.05^ab^ | 0.08±0.06^ab^ | 0.10±0.02^b^ |
| Sum | **6.54** | **6.81** | **6.50** | **6.55** | **5.98** | **2.97** |

Note: Data are average of three replicates ± standard deviations; “-”, not detected; Same lowercase letters in each line do not differ significantly at 5% probability by Duncan’s multiple range tests.

**Supplementary Figure legends**

**FIGURE S1:** Neighbor-joining phylogenetic tree based on 26S rDNA gene sequence of strain Y3401 and its closest relative species.

**FIGURE S2:** The change of remaining reducing sugar (a), fermenting property (b), and concentration of ethanol (c) in different fermentation with different inoculation methods. Black square, single-culture fermentation by *S. cerevisiae* Y3401 (S); red circle, single-culture fermentation by *W. anomalus* Y3604 (W); blue triangle up, simultaneous mixed fermentation was performed by inoculating 1×10^6^ CFU/mL of *S. cerevisiae* Y3401 and *W. anomalus* Y3604 (SMF); green diamond, inoculating 1×10^6^ CFU/mL of *S. cerevisiae* Y3401 for 12 h firstly, then 1×10^6^ CFU/mL of *W. anomalus* Y3604 was added (S-W); violet triangle down, inoculating 1×10^6^ CFU/mL of *W. anomalus* Y3604 for 12 h firstly, then 1×10^6^ CFU/mL of *S. cerevisiae* Y3401 was added (W-S). Results are the average and bars indicate the SD.

**FIGURE S3:** The change of remaining reducing sugar (a), fermenting property (b), and concentration of ethanol (c) in different fermentation with different inoculation ratio. Black square, inoculation ratio of *S. cerevisiae* Y3401 and *W. anomalus* Y3604 is 6:1; green star, inoculation ratio of *S. cerevisiae* Y3401 and *W. anomalus* Y3604 is 3:1; blue triangle up, inoculation ratio of *S. cerevisiae* Y3401 and *W. anomalus* Y3604 is 1:1; purple triangle down, inoculation ratio of *S. cerevisiae* Y3401 and *W. anomalus* Y3604 is 1:2; green diamond, inoculation ratio of *S. cerevisiae* Y3401 and *W. anomalus* Y3604 is 1:3; red circle, single-culture fermentation by *W. anomalus* Y3604. Results are the average and bars indicate the SD.

*Saccharomyces cerevisiae* strain VRI (KT222662.1)

*Saccharomyces cerevisiae* strain YI59 (KX428530.1)

*Saccharomyces cerevisiae* strain YI19 (KX428529.1)

*Saccharomyces cerevisiae* isolate KDLYS9-16 (KX119943.1)

**Y3401**

*Saccharomyces cerevisiae* (KX792963.1)

*Saccharomyces cerevisiae* isolate yazhong 6 (KX119944.1)

*Saccharomyces cerevisiae* isolate D3-2 (KP768092.1)

*Saccharomyces cerevisiae* strain NS-G-54 (KT923022.1)

*Saccharomyces cerevisiae* G40-11-19-1 (LC170447.1)

*Saccharomyces cerevisiae* strain LTQB31 (KR014239.1)

*Saccharomyces cerevisiae* strain V3 (KX428526.1)

64

64

31

64

0.0002

**FIGURE S1**





(a)





(b)





(c)

**FIGURE S2**





(a)





(b)





(c)

**FIGURE S3**
